# Automated pneumatic tourniquet for hemorrhage control evaluated using an adaptive testing platform

**DOI:** 10.3389/fmedt.2026.1844569

**Published:** 2026-06-17

**Authors:** Theodore G. Winter, Carlos Bedolla, Isiah M. Mejia, James P. Collier, Eric J. Snider

**Affiliations:** Expeditionary Medical Systems Department, US Army Institute of Surgical Research, Organ Support and Automation Technologies, Ft. Sam Houston, TX, United States

**Keywords:** automation, control system, medical device, tissue phantom, tourniquet

## Abstract

Hemorrhage remains a leading cause of preventable death in trauma situations with hemorrhage control being a critical first step to stabilizing injuries. Tourniquets are commonly used for this purpose, but they can adjust during transport leading to re-hemorrhage or tissue damage during prolonged use. Here, we explored control system approaches to automate tourniquet initial inflation and prolonged management. Different designs were compared and an approach was identified capable of hemorrhage control in under 40 s. Furthermore, automated control of the tourniquet successfully managed tourniquet loosening and overtightening situations that typical tourniquets could not manage. In summary, automated tourniquets have the potential to aid with hemorrhage control in early echelons of care while lessening the cognitive burden on limited medical personnel.

## 1 Introduction

Uncontrolled hemorrhage is the leading cause of preventable death in both military and civilian settings (1). To help mitigate the effects of hemorrhage, the “Stop The Bleed” initiative was implemented and has been successful. Individuals are trained through this program to quickly and effectively apply treatment at the point of injury to limit further blood loss until further medical intervention in a hospital setting can be provided (2–4). In a combat setting, extremity tourniquets are a common method for treating traumatic hemorrhage and are tools typically included in combat medic kits due to their small size and relatively simple use (2, 4, 5). Circumferential pressure is applied by the tourniquet to the limb to occlude arterial flow, resulting in reduced hemorrhage distal to the tourniquet. Current tourniquet types use different mechanisms to achieve vessel occlusion: (i) mechanical tourniquets use either a windlass rod or ratcheting system to apply circumferential tension and (ii) pneumatic tourniquets utilize inflatable cuffs to apply uniform circumferential pressure.

While these traditional devices are effective for initial hemorrhage control, there is an unmet need related to prolonged tourniquet use. This shortcoming has been magnified by recent global conflicts such as the Ukraine-Russia war, where prolonged application of tourniquets can result in tissue damage and necessary limb amputation (6). Conversely, tourniquet slippage is common during patient transport resulting in reaggravation of hemorrhage that may be unnoticed by medical providers. Additionally, the cognitive burden of tourniquet use alongside other battlefield stressors can result in improper use of tourniquets. Recent studies have reported that only 24.6% of tourniquet applications were appropriate (7). The low success rate is correlative to shortcomings in currently deployed tourniquets which do not confirm vessel occlusion or real-time assessments of the patient's dynamic conditions. These mechanisms can aid with avoiding under or over tightening the tourniquet to mitigate resulting rebleeds and tissue damage, respectively. Automated solutions are available in surgical settings and are effective at maintaining vessel occlusion for a prolonged period, but are often expensive, cumbersome, and not deployable in a fieldable, first responder environment (8). These limitations highlight the need for an improved adaptive tourniquet system.

The use of physiological closed-loop control (PCLC) systems can address the key limitations in tourniquet technology. PCLCs utilize real-time physiological signal sensing and algorithm controls to drive system actuation or activation to automate interventions (9). PCLCs have been used in multiple facets of patient hemorrhage management. For fluid resuscitation, PCLC use-cases are focused on utilizing mean arterial pressure (MAP) signal to drive infusion rates to reach a target blood pressure (10–14). For hemorrhage control, PCLCs have been implemented in the form of self-tightening and self-managing tourniquets that use sensors to provide constant monitoring and drive automated occlusion. For instance, tourniquets have been developed to utilize gears to drive automated tightening (15). Our research team has previously developed a proof-of-concept pneumatic tourniquet device that inflated by 15–30 mmHg steps until limb occlusion was achieved (16). However, active perturbances to the device after initial occlusion were not robustly evaluated and the PCLC for tourniquet inflation was slower than standard of care to achieve occlusion. In this work, we expand on these previous findings by evaluating different approaches to accelerate initial hemorrhage control and integrating an active arterial pulse monitoring algorithm that allows for the pressure control algorithm to modify internal cuff pressure. Real-time monitoring assessed how the automated pneumatic tourniquet (APT) re-tightened in the event of tourniquet slippage or pressure reduction due to over-pressure onset which could be expected if the tourniquet is forced higher on a thicker part of an extremity.

## 2 Materials and methods

For this work, the APT was designed with the intent to inflate to the point of arterial occlusion upon application. The detection of existing hemorrhage is based on the change of cuff pressure from arterial pulses, where consistent spikes in cuff pressure is evidence of arterial pulses, and, therefore, ongoing hemorrhage. The absence of such spikes results in a constant cuff pressure signal, providing evidence of zero flow going through this extremity. This design was tested and verified on a gel-limb phantom perfused with water, mimicking the anatomy of a human's extremity with an ongoing hemorrhage. The APT's functionality on this testing platform was compared against other commercially available tourniquets with the intent of illustrating comparable efficacy.

### 2.1 APT development

The APT was developed by integrating a Color Cuff® Size 18 inch (Stryker, Portage, MI, USA) into a data acquisition system that can (i) monitor pressure of the cuff for arterial pulses and (ii) drive a pump and solenoid system that controls airflow into the pressure cuff, driving occlusion of the extremity where the cuff is applied. To accomplish this task, Tygon LFL Tubing was connected from the pressure cuff to a pump and solenoid to control air intake and exhaust while another line was connected to a pressure sensor (015PGAA3, Honeywell, Charlotte, NC, USA) to monitor pressure inside the cuff. The analog output of the pressure sensor was read by a LabJack U3-HV (LabJack, Lakewood, CO, USA) before being converted to millimeters of mercury (mmHg) as a more appropriate unit of measurement using (1):

$$\mathrm{Pressure}\;(\mathrm{mmHg})=V_{\mathrm{in}}\;*\;5\;*\;775.724\;/\;0.8\;*\;5$$ (1)

Where V_in_ is the voltage output from the pressure sensor to an analog input pin on the LabJack, 5 represents the max voltage the pressure sensor can output when supplied with 5 volts, 775.724 is the maximum pressure the sensor can read in mmHg, and 0.8 is a constant value specific to this pressure sensor.

**Figure 1:**
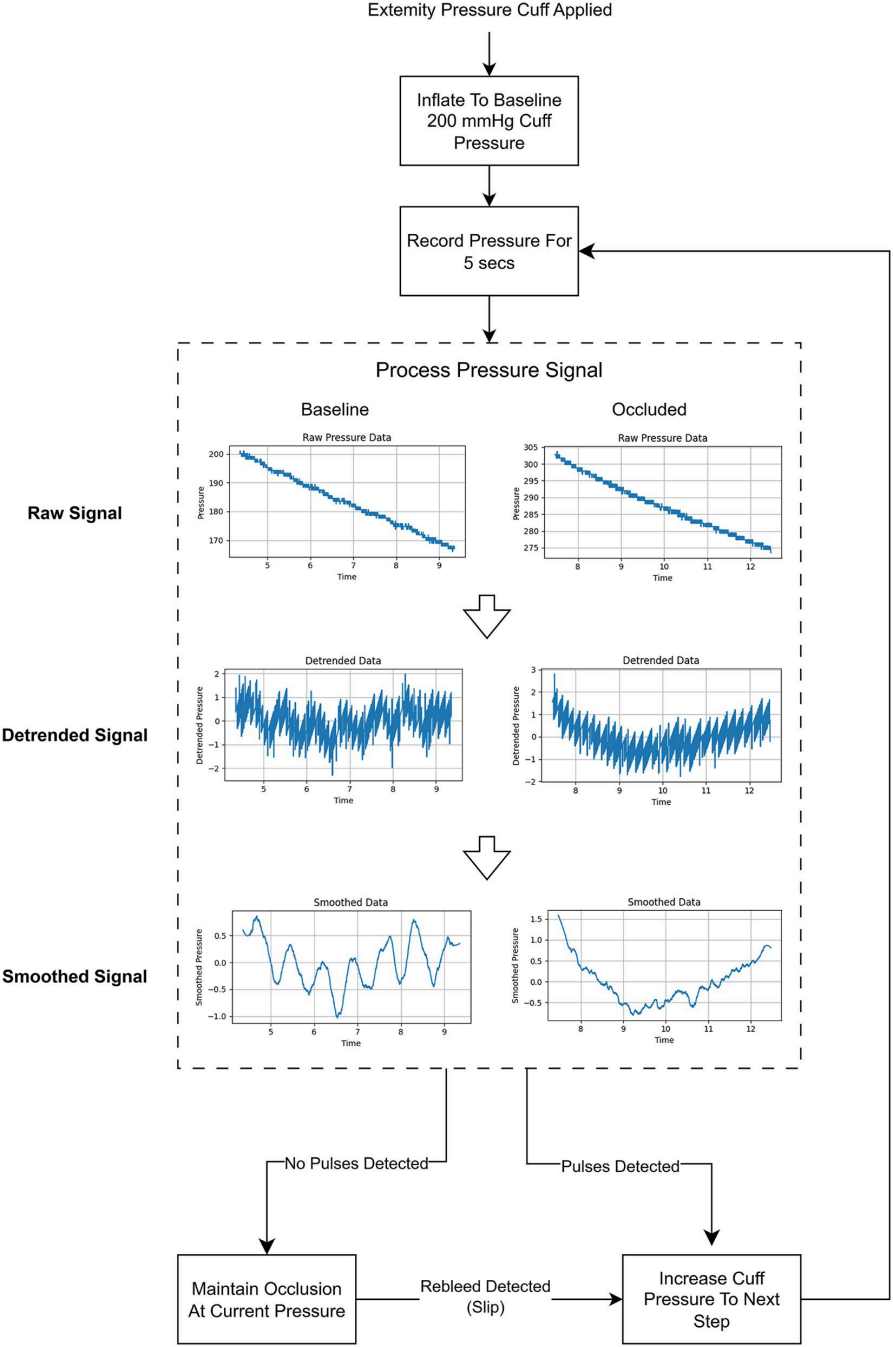
Diagram of the automated pneumatic tourniquet (APT) closed-loop control logic and the signal processing techniques utilized in its application.

To begin occlusion, first the sensor captures the ambient pressure created upon application of the cuff and uses this reading to offset later pressure readings to better represent how much the cuff has been inflated. The tourniquet is then inflated to a baseline pressure before incrementally inflating up to either occlusion of the extremity or a max cuff pressure of 360 mmHg based on algorithms described below. The pressure signal at each incremental step is captured for 5 s and monitored for arterial pulses. Processing of the raw pressure signal was inadequate as continuous pressure leakage and sensor susceptibility to high frequency noise did not yield a waveform from which arterial pulses could be intuitively derived. To fix this, a couple of signal processing techniques were implemented to make signal changes corresponding with arterial pulses more obvious. First, the signal was processed through a linear detrend transformation (17) to negate the negative slope of the pressure signal due to the continuous leak from the arm cuff. Second, a Savitsky-Golay filter (18) with a polyorder of 2 and a window length of 21 was applied to the detrended signal to remove any unwanted noise that deviated from the flow signal. Different combinations of polyorders and window lengths for the Savitsky-Golay filter were tested, and it was determined that the combination of a low polyorder and higher window length was able to remove most unwanted noise while preserving the general behavior of the signal. Once transformed, the pressure signal's peaks correlating to flow rate were much more obvious, making occlusion detection much more consistent (Figure 1).

Three different algorithms were implemented to determine cuff pressure behavior as it trended toward vessel occlusion of the application site. The first, which we refer to as Low-Baseline, initially inflates to a baseline pressure of 200 mmHg upon tourniquet application before inflating by 20 mmHg incremental steps until either occlusion is detected or the cuff reaches a maximum pressure of 360 mmHg. The next algorithm, referred to as High-Baseline, behaved similarly to Low-Baseline except it inflated to a higher initial pressure of 260 mmHg to try and bypass the initial pressure steps that the other approach would experience. The third algorithm, referred to as Adaptive-Steps focuses on the size of the incremental steps the APT increases its pressure by rather than the constant 20 mmHg increase to occlusion that the other two methods operate with. The process begins by inflating to a baseline pressure of 200 mmHg, however each incremental step value after this point was determined by the ratio of pulses detected (a max amount of 6 pulses per 5 s capture window) and 50% of the difference between the max pressure and the current pressure value. This increase is represented by the following (2):

Increase=(pulses/6)*0.5*(360-currentPressure) (2)

Where Increase is the amount of pressure the APT increases by, pulses is the number of arterial pulses detected over the 5 s window (maxed out at 6), 6 is the expected maximum number of pulses over that window, 0.5 is the factor to be applied to the difference between 360, the maximum pressure the APT is able to inflate to, and currentPressure, the pressure that the APT is currently inflated to. With this logic, the APT inflates up to half the amount of pressure needed to reach the maximum amount of pressure when 6 or more arterial pulses are detected. This incremental value respectively decreases as the number of pulses detected decreases.

### 2.2 Testing platform and testing scenarios

To test the APT, a testing platform was developed consisting of a tissue phantom, an expanding bone, and flow loop configuration. First, the phantom limb was created from a 1:5 mixture of EcoFlex 30 and EcoFlex 10 (Smooth-On, Inc., Macungie, PA, USA). The silicone mixture was poured into a cylindrical 3D printed mold (100 mm ID) which contained a concentric inner cylinder (48 mm OD) and a cylindrical channel (6.25 mm OD) placed approximately 6 mm from the mold wall, allowing for retrospective insertions of a rigid bone and silicone tubing to mimic arterial blood flow. The silicone was allowed to be cured for 6 h and removed from the mold.

**Figure 2:**
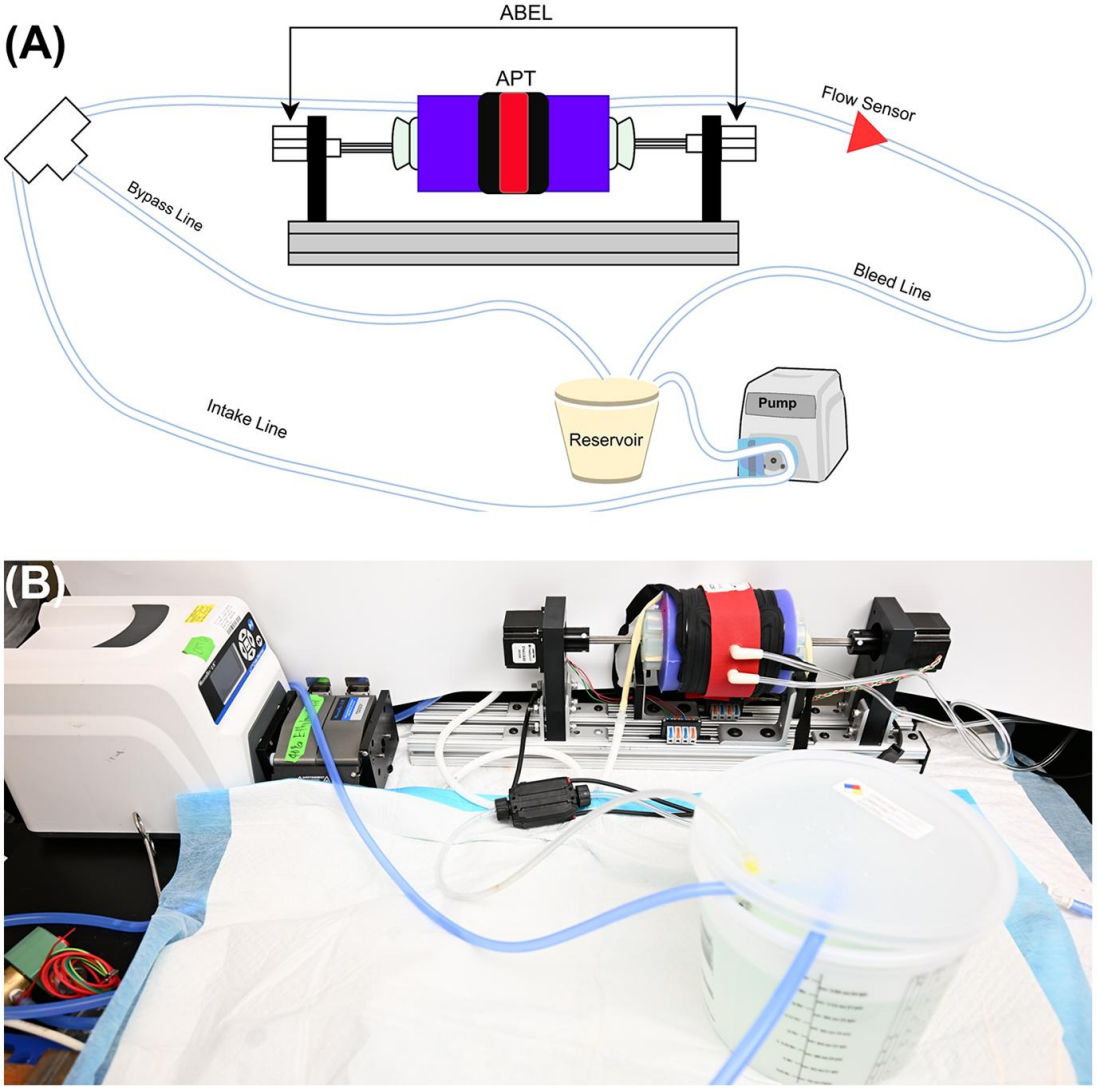
**(A)** Diagram of the adaptive testing platform utilizing a peristaltic pump to drive water through a phantom limb. **(B)** Image of the test platform setup. The phantom simulates slip by the cones moving through the collet sleeve embedded into the phantom. Arterial pulses are detected by the Automated Pneumatic Tourniquet (APT), which inflates and applies compressive force to the point of occlusion, cutting off flow down-stream of the phantom.

In addition to being able to maintain occlusion of the phantom limb for a prolonged period of time, the APT also needs to be able to adjust cuff pressure to accommodate for a scenario where the tourniquet may either slip and lose contact with the extremity or be forced further up where the pressure cuff becomes overtightened on the subject. To achieve this, the Artificial Bone for Expansion or Loosening (ABEL) was developed as shown in Figure 2. An expanding collet sleeve made from Durable Resin (Formlabs, Somerville, MA, USA) approximately seven inches in length and one inch in diameter serves as the expanding bone. This was inserted into the inner cavity of the phantom. The phantom and bone were suspended in between two cones, also made from the same Durable resin, attached to NEMA 17 stepper linear actuators and then placed at either end of the expanding collet sleeve. This is so that when the actuators extend, the Resin cones are shoved into the collet which expands the further they are pushed. This resin was chosen for its especially low coefficient of friction and its ductility without compromising toughness. With this system, we are able to test different bone sizes throughout the APT testing and simulate tourniquet slippage and overtightening.

Lastly, a flow loop was prepared to simulate flow from an open bleed using a gel-tissue phantom, as shown in Figure 2. Briefly, water perfusion was achieved by placing a ¼ inch Penrose tube in the artery channel of the phantom and perfusing water using a peristaltic pump (MasterFlex, Gelsenkirchen, Germany) configured with a dual head. Proximally to the arterial line, we connected a bypass line for fluid diversion when occlusion was achieved and a pressure sensor (Drager, Lubeck, Germany) to monitor and maintain physiological pressures. The distal arterial line was open to the intake reservoir to simulate bleed, and a flow sensor (FD-XA2, Keyence, Osaka, Japan) was used to capture the flow rate. MAP was maintained around 75 mmHg by adjusting the pump flow rates and fluidic resistance in the bypass and bleed lines using a tube clamp and blunt needle gauges.

The testing platform was used to test the APT and compare its performance to commercially available tourniquets: windlass tourniquet (Combat Application Tourniquet) and a manual pneumatic tourniquet (Color Cuff®). The testing platform was configured into three different ABEL expansion scenarios to test occlusion maintenance during slippage and over-tightening. For the purposes of this study, we defined vessel occlusion by a visual drip stop and a flow rate reduction of 95%. For the first scenario, ABEL was expanded to a maximum diameter and tourniquets were applied to maintain occlusion for 10 min. For the second scenario, ABEL was expanded to its maximum diameter and each tourniquet was applied and maintained occlusion for 5 min. A slip was then simulated by retracting ABEL's linear actuators and reducing the diameter of the artificial bone, inducing a re-bleed. The tourniquets were then retightened and maintained occlusion for 5 min. For the last scenario, the tourniquets achieved occlusion for 5 min with ABEL configured to its compressed configuration. ABEL was then expanded to simulate overtightening. Two force sensitive resistors (FSR) were embedded between the tourniquet and phantom to capture the force increase due to tourniquet overtightening. Each tourniquet was tested for three replicates (*N* = 3) for each scenario. The FSRs were calibrated using a Mark-10 universal tensile testing machine. For consistency, tourniquet application was conducted by a single experienced user for all the testing scenarios. Data was captured through a data acquisition system interfaced with LabChart 8 (ADInstruments, Sydney, Australia).

### 2.3 Statistical analysis

Testing results were conducted in at least three replicates for each tourniquet configuration, with primary results being time to achieve initial occlusion, total blood loss across the testing scenario, and overall force applied by the tourniquet. Times to reach occlusion were compared for statistically significant differences between APT logics and manual approaches. Overall total blood loss at the end of each scenario were compared between windlass, pneumatic, and APT approaches. For the overtightening scenario, force applied at the end of the scenario, relative to the force prior to overtightening were compared between manual approaches and the APT. Throughout, statistical differences were assessed by ordinary one-way analysis of variance (ANOVA) *post-hoc* Tukey's test. Significance was considered for *p*-values below 0.05 and are indicative in the results where appropriate.

## 3 Results

We first compared occlusion algorithms for the APT to determine which was most effective at reaching occlusion. Occlusion was determined to be when flow had been reduced to 95% of its original value prior to tourniquet application. All three of the APT's occlusion algorithms were tested against two commercially available extremity tourniquets for time to reach occlusion, amount of fluid lost upon reaching occlusion, and an increase in tourniquet compressive force upon overtightening.

**Figure 3:**
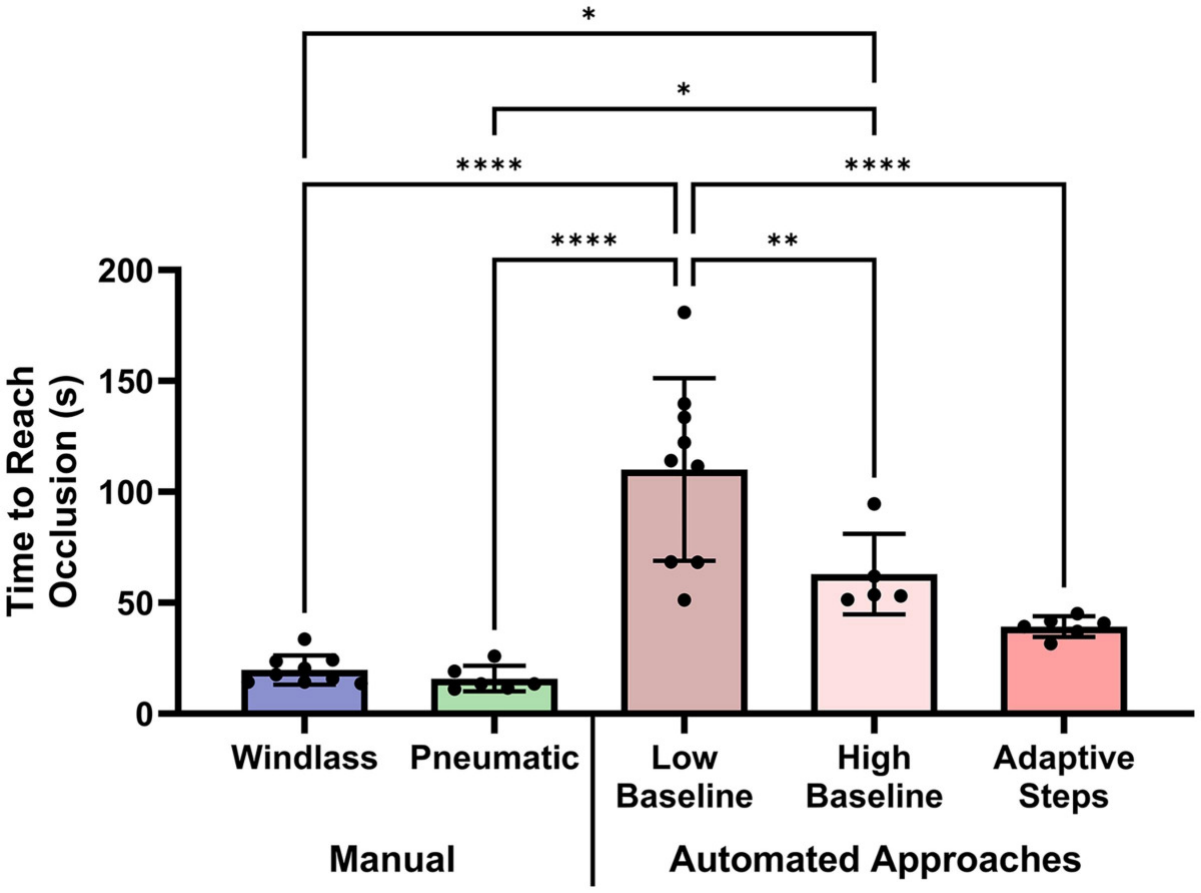
Comparison of occlusion times between manual tourniquet application and automated algorithms using the adaptive testing platform. Two different manual approaches were uses – (i) Windlass and (ii) Pneumatic – while three automated algorithms were evaluated – (i) Low-Baseline, (ii) High-Baseline, and (iii) Adaptive-Steps. Results are shown as mean, with error bars denoting standard deviation. Statistically significant differences between approaches were determined by Ordinary one-way ANOVA, post-hoc Tukey's test (* *p* < 0.05; ** *p* < 0.01; **** *p* < 0.0001).

Overall, each manual tourniquet was faster at achieving flow-stop occlusion compared to all occlusion algorithms tested with the APT, with the fastest being the manual pneumatic tourniquet which averaged 15.8 ± 5.7 s (Figure 3). Despite being the second fastest, the windlass tourniquet was prone to slipping during the prolonged period of time where each tourniquet was supposed to hold occlusion, with the tourniquet falling off the phantom in multiple testing runs. The different APT approaches were slower due to the feedback mechanisms required to prevent overtightening, with the fastest being Adaptive-Steps which inflated the cuff based on the number of arterial pulses detected during its 5 s arterial pulse capture window, averaging 39.3 ± 4.6 s; Adaptive-Steps logic was not significantly different compared to the windlass (*p* = 0.488) or manual pneumatic (*p* = 0.396) approaches, highlighting its stronger performance. This was also the most consistent method in the number of steps it took to reach occlusion on our testing platform, reducing the number of false occlusion detections prior to flow stop that occurred in the other two algorithms. The slowest approach was the Low-Baseline methodology wherein pressure was increased by 20 mmHg after initially reaching a 200 mmHg internal pressure, with an average time to reach occlusion of 110 ± 41 s; this approach was significantly slower than Adaptive-Steps (*p* < 0.0001), windlass (*p* < 0.0001), and manual pneumatic (*p* < 0.0001) This method also had the most inconsistent time to reach occlusion as it was especially prone to false occlusion detections when arterial flow was still present.

**Figure 4:**
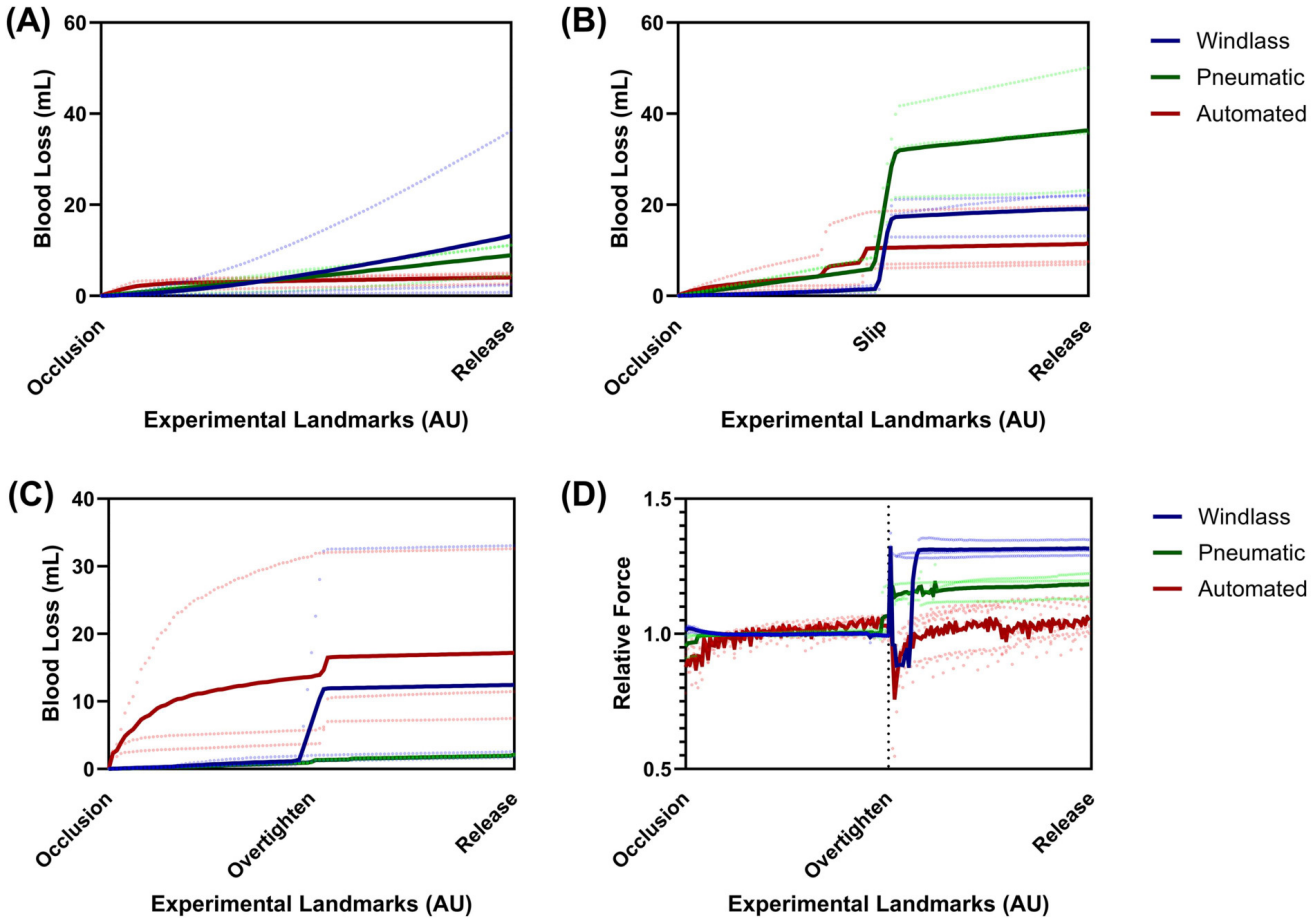
Comparison of performance for **(A)** normal, **(B)** slip, and **(C,D)** overtighten scenarios for windlass, manual, and automated tourniquets. **(A–C)** Blood loss after occlusion for each approach and **(D)** relative force after overtightening; force values are shown normalized to force at occlusion.

The APT and Manual Pneumatic tourniquets were successful at holding occlusion for a prolonged period of time (t = 10 min) and resulted in very little bleeding upon reaching drip-stop occlusion (Figure 4A). There were no significant differences in total hemorrhage volume between the APT (mean = 4.06 mL) and windlass (13.2 mL, *p* = 0.633) or pneumatic (8.93 mL, *p* = 0.872) approaches. The windlass tourniquet, as highlighted earlier, experienced a slip while holding occlusion during one of its runs while testing for the occlusion maintenance test runs, and correspondingly has a higher variance in flow lost from the re-bleed upon the slip. The APT was faster at responding to a slip from the bone causing a re-bleed, with it losing the smallest amount of fluid relative to the other two tourniquets (Figure 4B). In this scenario, the manual pneumatic tourniquet took the largest amount of time to adjust for the slip and lost the most amount of fluid among all tourniquets. APT (mean = 11.6 mL) had significantly reduced hemorrhage volume compared to the pneumatic for the slip scenario (36.3 mL, *p* = 0.039), but the windlass comparison was not significantly different (19.1 mL, *p* = 0.136). For the over-tightening runs, while the APT lost the most total amount of fluid, the windlass tourniquet had slipped off the phantom when the bone was expanded, causing a significant jump during the occluded period (Figure 4C). However, differences in total bleed volume were not significant between APT (mean = 17.1 mL) and the windlass (12.5 mL, *p* = 0.897) or pneumatic (2.11 mL, *p* = 0.386) approaches. As evidenced from the force data, the windlass tourniquet experienced the largest increase in force being driven into the phantom, while the APT experienced the smallest change in pressure when the bone expanded (Figure 4D). APT (mean relative force = 1.05) had significantly reduced force following overtightening compared to windlass (1.32, *p* = 0.0013) and pneumatic (1.18, *p* = 0.035) approaches.

## 4 Discussion

Extremity hemorrhage control requires refinement to minimize blood loss, particularly in a battlefield scenario where environmental conditions could either cause inadequate placement of a tourniquet or tourniquet failure after application. Such failure can cause the tourniquet to lose contact to the point of allowing a previously treated hemorrhage to re-bleed. Alternatively, the possibility of tourniquet overtightening can lead to tissue necrosis and re-perfusion injury effects beyond the hemorrhage of tourniquet slippage. As it stands, the deployment of traditional extremity tourniquets requires direct monitoring by a medical professional to limit the potential harm that could come from tourniquet slippage or overtightening, especially during prolonged application of a tourniquet which may not be available during combat casualty care.

The APT developed in this work helps solve this problem through the active monitoring of arterial blood flow, where it inflates to the point of eliminating the detected arterial pulses and active hemorrhage. Upon achieving occlusion, the APT is able to continuously monitor changes to internal pressure that could be from either the tourniquet slipping or overtightening and modify its pressure accordingly, maintaining occlusion without overtightening significantly past the point of bleed stop. By doing so, the cognitive burden that comes with tourniquet application in a high stress scenario is significantly decreased. Additionally, the automatic monitoring for changes to cuff pressure eliminates the need to directly watch for traditional tourniquet failure, as the APT will adjust according to internal arterial pulse detection without human intervention.

While the APT is successful at eventually reaching and maintaining occlusion, limitations of the APT's current design mostly stem from the inadequate application time and the time it takes to reach occlusion. While different methodologies of reaching occlusion were implemented with some performing significantly better than others, even the best APT inflation logic was not as fast as either commercially available manual tourniquet. It is important to note that manual tourniquet application was performed by someone with extensive knowledge from prior tourniquet research, and an individual with less experience could see a higher or less consistent time to reach occlusion. At a bare minimum, the APT could be configured for initial manual hemorrhage control that was routinely quicker than the automated approaches, followed by pressure control by the automated algorithms to maintain proper hemorrhage control. Additionally, the results of this study showed some variance for each tourniquet device tested. Since testing was conducted in a controlled testing platform, limited amount of variance should be seen. Thus, the difference between occlusion time and total blood loss volume could be a result of user error, over or under tightening, when manually applying the tourniquets. Due to the prototype nature of the APT, the configuration connecting the pump, solenoid valve and pressure sensor could lead to some degree of air leakage, making blood loss volume inconsistent.

The APT was also prone to early occlusion detection, where the logic for detecting arterial pulses would determine flow stop despite a bleed being present, leading to an adjustment period where the APT would have to detect that a rebleed was present before inflating to additional pressure steps, increasing the time to achieve occlusion. Although the third occlusion method was significantly more consistent in the time it took to reach vessel occlusion, the pressure sensor's susceptibility to noise could drown out any pulses coming from the extremity, triggering early occlusion detection while a hemorrhage is still present.

These aspects of the APT represent shortcomings that need to be improved prior to deployment. Such solutions revolve around improved signal analysis of internal cuff pressure of the APT, where different signal processing or filtering techniques could be employed to consistently detect arterial peaks. Another solution could involve moving away from a monitor-by-step algorithm that was used in this work for one that monitors a limb's occlusion level as the cuff is inflating, reducing the tourniquet's time to reach occlusion by removing the 5 s window in between each pressure step that attempts to correlate peaks in pressure readings to arterial pulses. Additionally, a lighter and reduced footprint of the circuitry that was used for the APT could help make the device more transportable and more likely to be carried in a real-life scenario. The relevant durability and field testing should accompany this potential future effort.

## 5 Conclusion

In conclusion, the APT shown in this work was tuned to quickly achieve bleed stop in less than one-minute timeframes. Furthermore, the APT control logic could manage overtightening and tourniquet slippage effects that typical manual tourniquets could not mitigate. Ultimately, this provides proof-of-concept evidence of PCLCs for this hemorrhage control application which potentially can improve mortality outcomes during trauma. Next steps will evaluate this technology in more translatable testing platforms such as live animal models to show efficacy for prolonged durations where PCLCs can most improve tourniquet application.

## Data Availability

The datasets presented in this article are not readily available because they have been collected and maintained in a government-controlled database located at the U.S. Army Institute of Surgical Research. This data can be made available through the development of a Cooperative Research and Development Agreement (CRADA) with the corresponding author. Requests to access the datasets should be directed to Eric Snider, eric.j.snider3.civ@health.mil.
